# Analysis of scintillation light intensity by microscopic radiation transport calculation and Förster quenching model

**DOI:** 10.1371/journal.pone.0202011

**Published:** 2018-08-29

**Authors:** Tatsuhiko Ogawa, Tetsuya Yamaki, Tatsuhiko Sato

**Affiliations:** 1 Research Group for Radiation Transport Analysis, Division of Environment and Radiation Sciences, Nuclear Science and Engineering Center, Japan Atomic Energy Agency, Shirakata, Tokai, Ibaraki 319-1195, Japan; 2 Takasaki Advanced Radiation Research Institute, National Institutes for Quantum and Radiological Science and Technology, Watanuki, Takasaki, Gunma 370-1292, Japan; Universitat Zurich, SWITZERLAND

## Abstract

The scintillation light yield of plastic scintillator considering the quenching effect is reproduced by a calculation model based on a track-structure simulation code and the Förster effect. Energy deposition and its nm-scale spatial arrangement in the irradiation by electrons, protons, and heavy ions (^4^He to ^81^Br) in an NE-102A scintillator were simulated by a track-structure simulation code. The spatial arrangements of the excited molecules emitting scintillation light and those dissipating the excitation energy were then obtained to calculate the strength of the quenching effect. Light emission from the excited molecules was integrated to finally obtain the observable light yield. The calculated light yields are in good agreement with the earlier measurement data. Moreover, in the case of low-LET particle incidence, a statistical micro-dosimetric model can substitute the track-structure simulation code for reproducing the light yield.

## Introduction

Scintillators are indispensable for radiation detection in various fields. A great variety of scintillator-based detectors have been developed and applied to diverse fields, including radiation safety, medicine, industry, space science, and fundamental physics. Generally, scintillation light is converted to electrical signals by using devices such as photo-diodes or photo-multipliers. The intensity of these electric signals is then used to analyze dose, energy, and particle species.

The light output of scintillators is basically proportional to the amount of the energy deposited in the scintillator crystal but it is known that the light output is suppressed in case of high-LET(linear energy transfer) radiation such as low-energy protons and heavy ions owing to the quenching effect. As a result, the light yield is not linear to the deposited energy in the high-LET regime. Birks’ law [1] is the most common formalism to estimate the light output of scintillators exposed to protons and ions considering quenching. On the basis of experimental data, Smith [2] proposed a revision to the Birks’ law to reproduce the light yields in a wider LET range. These formulas assume that light output is described as a function of LET. The fitting parameters were estimated based on experimental data on scintillation light yield for various incident particles. Therefore, an experimental data-set large enough to determine the fitting parameters is necessary to predict light yields. Moreover, in the case of low-energy heavy-ion incidence, light output depends not only on LET but also on ion species [3]. In other words, the light yield by particles having the same LET can differ depending on the particle species. In that case, LET-based systematics such as Birks’ law cannot predict light yields. An approach based on quantities other than LET is therefore necessary to reproduce scintillation light output universally.

In the present study, we propose a new computational model for predicting the light outputs of scintillators exposed to various radiations. Basic assumptions of this model are 1: light output is attributed to the yield of excited fluorescent molecules (referred to as donors hereinafter) and 2: quenching is caused in by Förster resonance energy transfer (FRET) [4] between fluorescent molecules. To simulate the spatial arrangement of donors, which is important to estimate the strength of FRET, energy deposition in nm-scale by incident particles was tracked explicitly by using a track-structure simulation code.

An alternative model based on a systematic specific energy calculation model is also proposed for prediction of light output owing to low-LET radiation. Because most of the currently-available track-structure simulation codes and specific energy calculation models were developed for liquid water, this study focuses on plastic scintillators, whose atomic number and density are close to those of liquid water. Accuracy of these methods was tested against the measured light output of NE-102A scintillators.

## Methods

In this study, an NE-102A scintillator (equivalent to EJ-212), a common scintillation material used for radiation detection, was taken as an example. NE-102A is composed of polyvinyl-toluene (PVT), p-terphenyl (p-TP) and 1,4-Bis[2-(5-phenyloxazolyl)] benzene (POPOP). The mass fractions of these components are 97, 3, and 0.005, respectively [5]. In scintillator materials, the majority of incident radiation energy is used to ionize and excite the molecules of detector materials. The electrons recoiled by incident ions give their energy to secondary electrons by electron cascade. The cascade electrons are down-scattered gradually and are absorbed eventually by the scintillator molecules. Because NE-102A consists mainly of PVT, the energy deposited by incident radiation is received by PVT. The excited PVT molecules are referred to as primary donors hereinafter. The excitation energy of primary donors is transferred to the molecules of the fluorescent substance, which have a similar band gap as that of the primary donor molecules. In NE-102A, the excitation energy of some primary donors is absorbed by phonons (i.e., converted to thermal energy) or other donors whereas the rest is transferred to p-TP and POPOP, and is finally emitted from POPOP as photons. Thus the wavelength spectrum of the initial scintillation light was shifted owing to the Stokes shift of p-TP and that of POPOP. The wavelength of resulting light is longer than that of PVT absorption band; therefore, the light emitted by POPOP can reach the photoelectric surface of the detector with little absorption in the PVT matrix. Hence, the light yield is proportional to the number of excited POPOP molecules.

Birks [1] proposed that the non-linearity of the light yield against the deposited energy is attributed to the energy transfer to damaged molecules, which dissipate the received energy non-radiatively. Birks fitted the experimentally known light yield of scintillators as a function of LET,

$$\begin{array}{c} \frac{dL}{dx}=S\frac{\frac{dE}{dx}}{1+kB\frac{dE}{dx}}, \end{array}$$ (1)

where *S* is the normalization factor, *k* is the energy transfer probability, *B* is the constant of proportionality to determine the number of damaged molecules, and *dE*/*dx* is LET. The Birks’ formula is useful for explaining the light yield of scintillators in various conditions, however Matsufuji *et al.* [3] pointed out that the formula cannot be applied to high LET particles. This is because the Birks’ formula does not consider the spatial configurations of the excited fluorescent molecules and damaged molecules explicitly, whereas the strength of energy transfer is sensitive to the distance between them.

Therefore, in the present study, the strength of energy transfer was calculated based on the detailed spatial configuration of energy deposition. It is also known that the Dexter mechanism [6] and exciplex formation are responsible for the suppression of light yield. However, these mechanisms take effect between molecules in close contact with each other and are therefore considerably less significant than FRET. It should be also noted that the excitation energy is dissipated not only by inter-molecular interactions but by non-radiative intrinsic de-excitation of the excited molecules, but such intrinsic de-excitation was not considered because it does not depend on the spatial configuration of excited molecules and affects only the absolute light yield. Normally, scintillators are used in combination with amplifiers such as photo-multipliers or photo-diodes. Therefore, the absolute light yield or intrinsic de-excitation was not considered. It should be also noted that the number of donors is significantly less than the number of molecules. In case of 10 MeV ^81^Br ion incidence to liquid water, track structure calculation by RITRACKS code [7] showed that the ionization density in the track core with 10 nm of radius is 2.5 × 10^−2^ (/nm^3^) while the molecular density of PVT is 5.1 (/nm^3^). The probability that one molecule is ionized twice is 1.3 × 10^−4^. Even in the track core with 1 nm of radius, the ionization density is 0.59 (/nm^3^) and thereby the probability that one molecule is ionized twice is 6.9 × 10^−2^. These facts show that it is unlikely that energy is deposited on the same molecule more than once.

The probability of occurrence of FRET depends on the donor-acceptor distance [4], as expressed by Eq (2),

$$\begin{array}{c} p\left(r\right)=\frac{1}{1+{\left(\frac{r}{R_{f}}\right)}^{6}}, \end{array}$$ (2)

where *R*_f_ is a material-specific constant referred to as Förster radius, and *r* is the distance between the donor and the acceptor. In this study, it was assumed that the donors play the role of the acceptors. The reasons are as follows. The quenching effect in NE-102A is consistent between the earlier experiments, therefore it is unlikely that quenching is attributed to crystal defects, which differ from one detector to another. In addition, the presence of particular molecular states which is responsible for quenching (e.g., “damaged molecule” in Birks’ law) has not been indicated but a large amount of acceptors are necessary to reproduce the experimental data discussed later. Donors were assumed to play the role of acceptors by the following mechanism. Most of the PVT molecules excited by irradiation are at the first excited state (S_1_). If two PVT molecules at S_1_ state are close to each other, the excitation energy of one molecule can be transfered to another by FRET. Eventually, one molecule goes to the ground state (S_0_) and the other goes to a higher excited state such as S_2_ and then the molecules in the higher excited state are de-excited to S_1_ state. According to the Kasha’s rule [8], S_2_ → S_1_ transition is non-radiative; therefore, fluorescence photon is emitted mostly from S_1_ → S_0_ transition. Thus the energy of one donor is dissipated by a neighboring donor. The Förster radius of NE-102A (or equivalent EJ-212, BC-400) has not been measured, but the Förster radii of the organic molecules used in scintillation detectors range from 2 to 6 nm [9]. Therefore the Förster radius of NE-102A was assumed to be 4 nm in this study. The dependence of the light yield on the Förster radius is discussed later. In FRET analysis, the spatial arrangement of excited molecules, in the order of a few 10 nm, plays a critical role. To simulate the excitation of molecules at the nm scale and the quenching effect between the molecules, two methods are proposed as described below.

### Track-structure method

The first method is based on the energy deposition calculated by a Monte-Carlo track structure simulation code called RITRACKS [7]. RITRACKS traces each electron recoiled by incident radiation (e.g., ions, electrons and photons) by using cross sections down to a few eV until they are absorbed or stopped. The official release of RITRACKS Ver.3.06 can handle transport of ions up to Ni but scintillation by ions up to Br was analyzed in this study. Therefore, we used RITRACKS Ver. 3.1 [10], which can simulate ions up to Rn, in this study. By using RITRACKS, the spatial coordinates of energy deposition and the amount of the energy deposited at each point were obtained. RITRACKS implicitly assumes that the target material is water because the code was developed originally for radiation biology. Energy is deposited considering the modes such as excitation, molecular rotation, and ionization. Therefore interpretation is necessary to apply the calculated result to NE-102A scintillator. The density of NE-102A scintillators is 1.032 (g/cm^3^) whereas the energy deposition calculation by RITRACKS was done in water. Assuming that the spatial distribution of energy deposition can be scaled by the density, the (x,y,z) coordinates of energy deposition was scaled down by 0.990 (= 1/^3^ $\sqrt{1.032}$). Alternatively, the distance can be scaled by electron density. The electron densities of NE-102A and liquid water are 0.560 (mol/cm^3^) and 0.556 (mol/cm^3^), respectively; therefore, the scaling factor deduced from electron densities is 0.998. This value is close to the scaling factor deduced from the mass densities (0.990), which was adopted in this study. Owing to the mass density and electron density close to those of water, the results calculated for water can be translated to NE-102A but special attention must be paid when our method is applied to scintillators with density significantly different from that of water.

The matrix of an NE-102A scintillator is composed of polyvinyl toluene, the principal resonance wavelength of which is 330 nm (3.7 eV). This means that energy deposition smaller than 3.7 eV does not contribute to light yield. In addition, the dynamics of electrons in condensed matter is scarcely known therefore it is not clear which energy deposition reaction corresponds to donor production. Therefore ionization from the orbitals, 1b_1_ (8.76 eV), 3a_1_ (12.1 eV), 1b_2_ (16.8 eV), 2a_1_ (32.2 eV), 1a_1_ (40.0 eV), and dissociative electron attachment (DEA, in the range from 7 to 14 eV) was assumed to equivalently contribute to the production of a donor. By contrast, excitation, rotation, and vibration were disregarded in the calculation of light yield because energy deposition by the vibration and the rotation modes is too low to emit photons (generally lower than 1 eV). To verify this assumption, light yields were calculated by using the track-structure method, which is explained later, under some typical conditions with assuming that all energy deposition greater than 3.7 eV, regardless of the reaction mode, contributes to production of donors. The calculated light yield was compared with that calculated with the standard condition (i.e. only ionization greater than 8.76 eV contributes to production of donors). As shown in Table 1, the assumption affects the light yield by no more than 13% and normally by less than a few percent. The discrepancies for electrons, protons and GeV-class heavy ions are smaller than the fluctuation of experimental data as shown in “Results and discussion” section. The discrepancy for ^81^Br indicates that the light yield for 40 MeV ^81^Br might be underestimated by 13%. This discrepancy is discussed later.

**Table 1:** Effect of the energy deposition threshold adopted in the light yield calculation. The ratio was calculated by (light yield with 3.7 eV threshold)-(light yield with 8.76 eV threshold)/(Light yield with 3.7 eV threshold) * 100.

Incident particle		Ratio (%)
0.2 MeV	e^-^	0.052
1 MeV	e^-^	0.024
3000 MeV	^20^Ne	0.063
1 MeV	^1^ H	5.1
40 MeV	^81^Br	13
120 MeV	^81^Br	3.0

**Fig 1:**
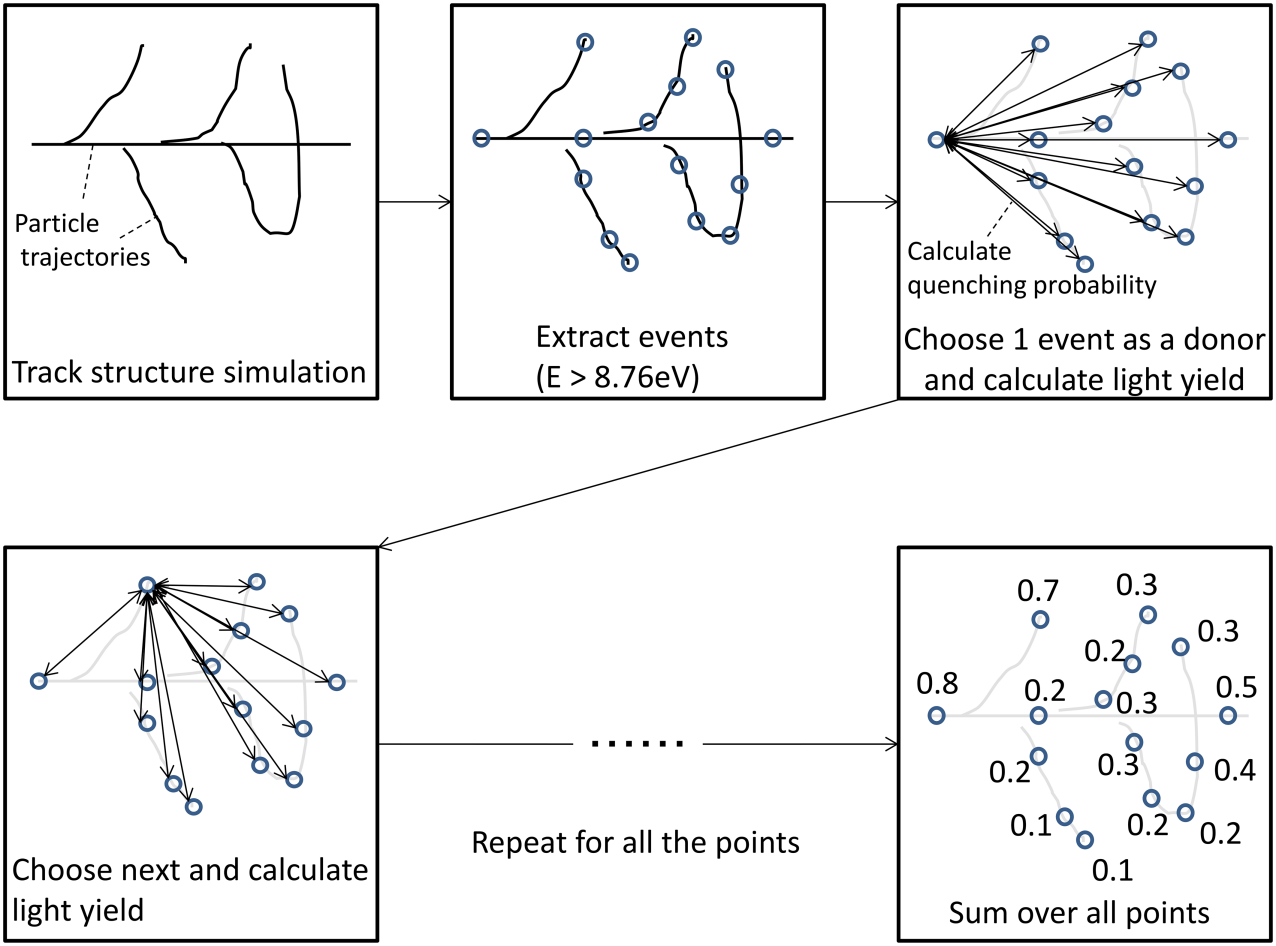
Calculation scheme of the track-structure method. Beam incident direction is defined as y axis.

The calculation scheme of the track-structure method is shown in Fig 1 and described below. After performing the track structure calculation using RITRACKS, the (x,y,z) coordinates and the energy deposited in each energy deposition event were recorded. To suppress statistical uncertainty, the RITRACKS calculation was continued until more than 500,000 events were recorded. To calculate energy deposition by energetic punch-through particles (particles with energy high enough to penetrate the scintillator), RITRACKS calculation was performed only at the incident energy because it is reasonable to approximate that the particle energy is constant throughout the particle track. By contrast, RITRACKS calculation was carried out at several energies in full-stop condition (the energy of incident particles is so low that they are stopped inside the scintillator) because the energy of the incident particles varied down to 0. At first, events were filtered based on their deposition energy (*E* ≥ 8.76 eV) and sorted by their y (incident beam axis) coordinates. The first energy deposition event was selected and the distances of the 2,000 closest events were calculated. The probability of FRET between the selected event (donor) and 2,000 of its neighboring donors was estimated using Eq (2). This donor was removed and not considered later if FRET takes place. Otherwise, the light yield was incremented. It was confirmed that the FRET probability depended on the number of considered neighboring donors (2,000 in this case) by less than 0.1%. Owing to the strong dependence of FRET on the molecular distance (See Eq 2), the intensity of FRET is dominantly determined by the 2,000 closest molecules. The next donor was then selected and the same calculation was performed. This calculation process was repeated for all subsequent donors.

### Specific energy method

The drawback of the track-structure method is the long computation time for track-structure simulation. In view of this, a less time-intensive method based on the parameterization of statistical specific energy distribution was developed. This method is referred to as the SE method hereinafter.

The T-SED function [11] of PHITS [12] is used to calculate microdosimetric quantities in liquid water exposed to radiation. Instead of tracking low energy electrons down to eV energy range, T-SED splits the water target into small spherical cells and calculates lineal energy (y) or specific energy (z) based on their parameterization for various incident particle species and energies. The radius of the spherical cells is defined by users.

In this study, z was calculated for various incident radiations by using T-SED. The radius of the spherical cells was 3.96 nm, which is equal to the Förster radius of NE-102A assumed in the track-structure method with correction for the density. The cell radius did not have to be equal to the Förster radius but substantial portion of FRET can be considered in this setup. In the same way as in the track-structure method, a donor was assumed to be produced each time 8.73 eV of energy was deposited in a cell. The number of donors in the cell was calculated as ⌊ (energy deposited in the cell)/ (8.73 eV) ⌋. Because PHITS does not track the trajectories of electrons even using T-SED, the spatial coordinates of energy deposition were lost. Therefore, it was necessary to estimate the strength of quenching statistically.

The probability that FRET occurs between two excited molecules in the sphere was calculated using Eq (2). The light yield *L* is proportional to the number of donors that are not suppressed by FRET. Therefore, it was calculated as

$$\begin{array}{ccc} & & L=\sum_{N=1}^{\infty }L_{N}\\ & & \;=\sum_{N=1}^{\infty }C_{N}l_{N}, \end{array}$$ (3)

where *C*_*N*_ is the number of cells containing *N* donors, which was calculated using T-SED, and *l*_*N*_ is the expected light yield from a cell in which the number of donors is *N*.

Because there were large numbers of cells in the system and donors were distributed at random, *l*_*N*_ was calculated by introducing an approximation: the coordinates of donors were distributed uniformly and independently from each other. The expected light yield can be obtained by averaging Eq (2) as,

$$\begin{array}{ccc} & & l_{0}=0,\\ & & l_{1}=1,\\ & & l_{2}=\frac{2}{{\left(4\pi r_{\text{se}}^{3}/3\right)}^{2}}\int^{v}\int^{v}p(|\vec{r_{1}}\left(r_{1},\theta_{1},\phi_{1}\right)-\vec{r_{2}}\left(r_{2},\theta_{2},\phi_{2}\right)\left|\right)d\vec{r_{1}}d\vec{r_{2}},\\ & & l_{3}=\frac{3}{{\left(4\pi r_{\text{se}}^{3}/3\right)}^{3}}\int^{v}\int^{v}\int^{v}p(|\vec{r_{1}}\left(r_{1},\theta_{1},\phi_{1}\right)-\vec{r_{2}}\left(r_{2},\theta_{2},\phi_{2}\right)\left|\right)\\ & & \;\;\;\;\;\;\times p(|\vec{r_{1}}\left(r_{1},\theta_{1},\phi_{1}\right)-\vec{r_{3}}\left(r_{3},\theta_{3},\phi_{3}\right)\left|\right)d\vec{r_{1}}d\vec{r_{2}}d\vec{r_{3}}, \end{array}$$ (4)

where ∫^v^ *dr*_*i*_ denotes integration over the spherical volume, and $\vec{r_{1}}$ and $\vec{r_{2}}$ denote the coordinates of the donors.

Because the coordinates of the spherical volumes were chosen at random, the donor’s coordinates were defined as the center of the cell (i.e., *r*_1_ = 0). Under this assumption, Eq (4) can be simplified as follows owing to isotropy,

$$\begin{array}{r} l_{2}=\frac{2}{4\pi r_{\text{se}}^{3}/3}\int_{0}^{r_{\text{se}}}4\pi r^{2}\left(1-\frac{1}{1+{\left(\frac{r}{R_{\text{f}}}\right)}^{6}}\right)dr\\ =\frac{2}{r_{\text{se}}^{3}/3}\left(\frac{r_{\text{se}}^{3}}{3}-\frac{R_{\text{f}}^{3}}{3}\text{ArcTan}\left[\frac{r_{\text{se}}^{3}}{R_{\text{f}}^{3}}\right]\right)\\ =2\left(1-\frac{R_{\text{f}}^{3}}{r_{\text{se}}^{3}}\text{ArcTan}\left[\frac{r_{\text{se}}^{3}}{R_{\text{f}}^{3}}\right]\right)\\ =\;0.429,\\ l_{3}=\frac{3}{4\pi r_{\text{se}}^{3}/3}{\left[\int_{0}^{r_{\text{se}}}4\pi r^{2}\left(1-\frac{1}{1+{\left(\frac{r}{R_{\text{f}}}\right)}^{6}}\right)dr\right]}^{2}\\ =\frac{3}{r_{\text{se}}^{3}/3}{\left(\frac{r_{\text{se}}^{3}}{3}-\frac{R_{\text{f}}^{3}}{3}\text{ArcTan}\left[\frac{r_{\text{se}}^{3}}{R_{\text{f}}^{3}}\right]\right)}^{2}\\ =\;0.414,\\ l_{m}=\frac{m}{4\pi r_{\text{se}}^{3}/3}{\left[\int_{0}^{r_{\text{se}}}4\pi r^{2}\left(1-\frac{1}{1+{\left(\frac{r}{R_{\text{f}}}\right)}^{6}}\right)dr\right]}^{m-1}\\ =\frac{m}{r_{\text{se}}^{3}/3}{\left(\frac{r_{\text{se}}^{3}}{3}-\frac{R_{\text{f}}^{3}}{3}\text{ArcTan}\left[\frac{r_{\text{se}}^{3}}{R_{\text{f}}^{3}}\right]\right)}^{m-1}, \end{array}$$ (5)

where *r*_*se*_ is the radius of the spherical volumes (3.96 nm in this case).

## Results and discussion

**Fig 2:**
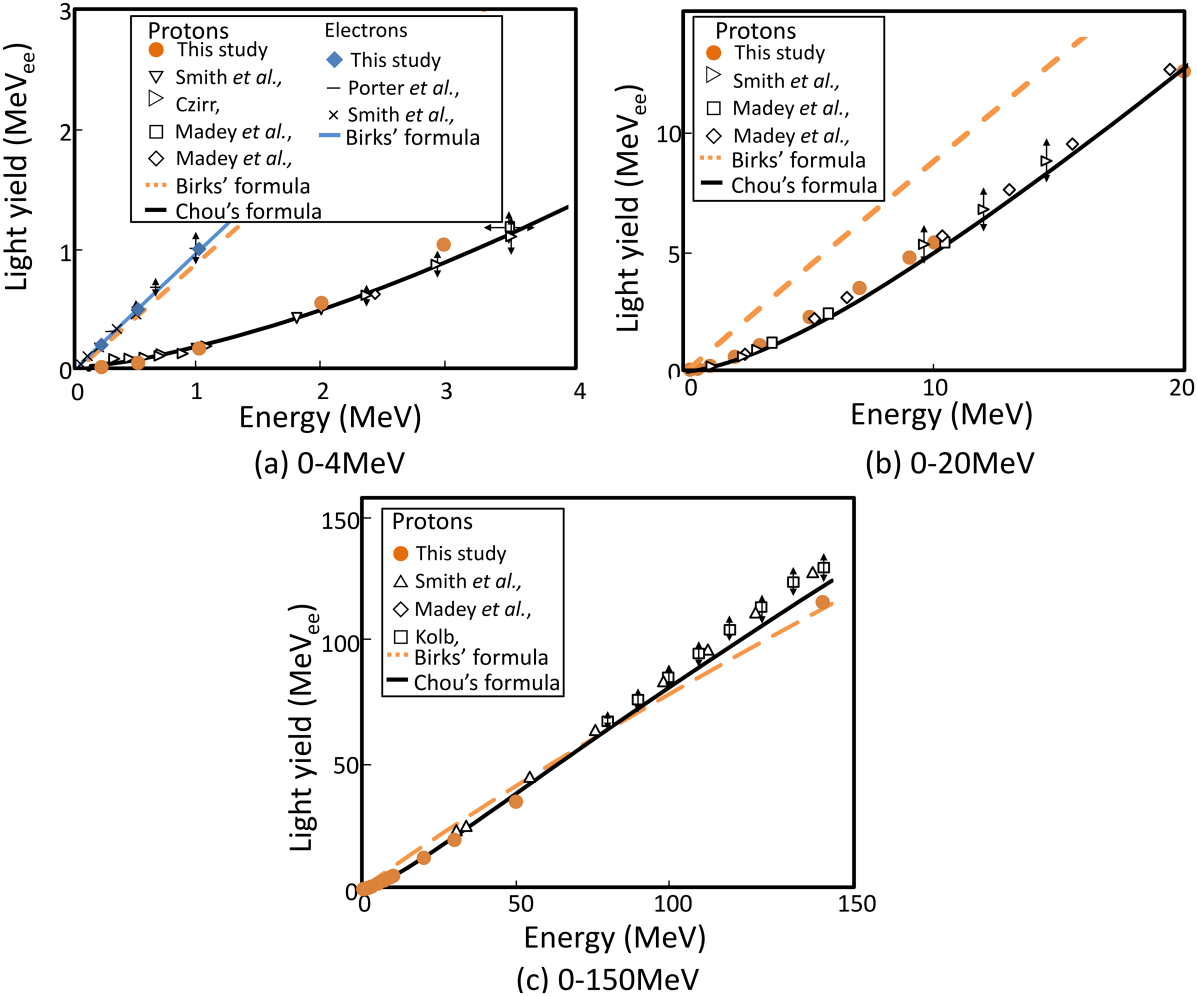
Light yields of NE-102A irradiated by protons and electrons of various energies. Simulation was based on the track-structure method. “This study” denotes the values obtained by the track-structure method. Dashed and solid lines denote fitting by Birks’ formula [1] and Chou’s formula [19], respectively. The other values are experimental data from the literature.

The light yield calculated by using the track-structure method was compared with earlier measurement data. Fig 2 shows the light yield of NE-102A irradiated by full-stop protons and electrons of various energies [13–18].

All calculated data were normalized for 1 MeV electrons. The intensity of scintillation light owing to 1 MeV electrons is expressed as 1 MeVee hereinafter. Both the calculated and the measured light yields due to electrons increase linearly with the incident energy because energy deposition by electrons is so sparse that quenching is weak. By contrast, the light yield due to protons is suppressed strongly by quenching. For example, the light yield due to 1 MeV protons is about a factor of 10 lower than that due to 1 MeV electrons owing to dense energy deposition by protons. In our calculation, both the linear light yield response by electrons and the nonlinear light yield by protons were reproduced accurately. Energy deposition density decreases with increasing incident proton energy and therefore the significance of quenching decreases. In Fig 2(b), light yield has an almost linear relationship with incident energy near 10 MeV. Fig 2(c) shows that the quenching effect is considerably less significant at high energies. The light yield due to protons has an almost linear relationship with incident energy. The Birks’ model reproduces the light yield by electrons and that by high-energy protons but strong quenching for low-energy proton irradiation was beyond its capacity. By contrast, the fitting by Chou’s model agrees well with the measurement data. Chou’s model can evidently reproduce the data for electrons by substituting the factor of (LET)^2^ with zero.

**Fig 3:**
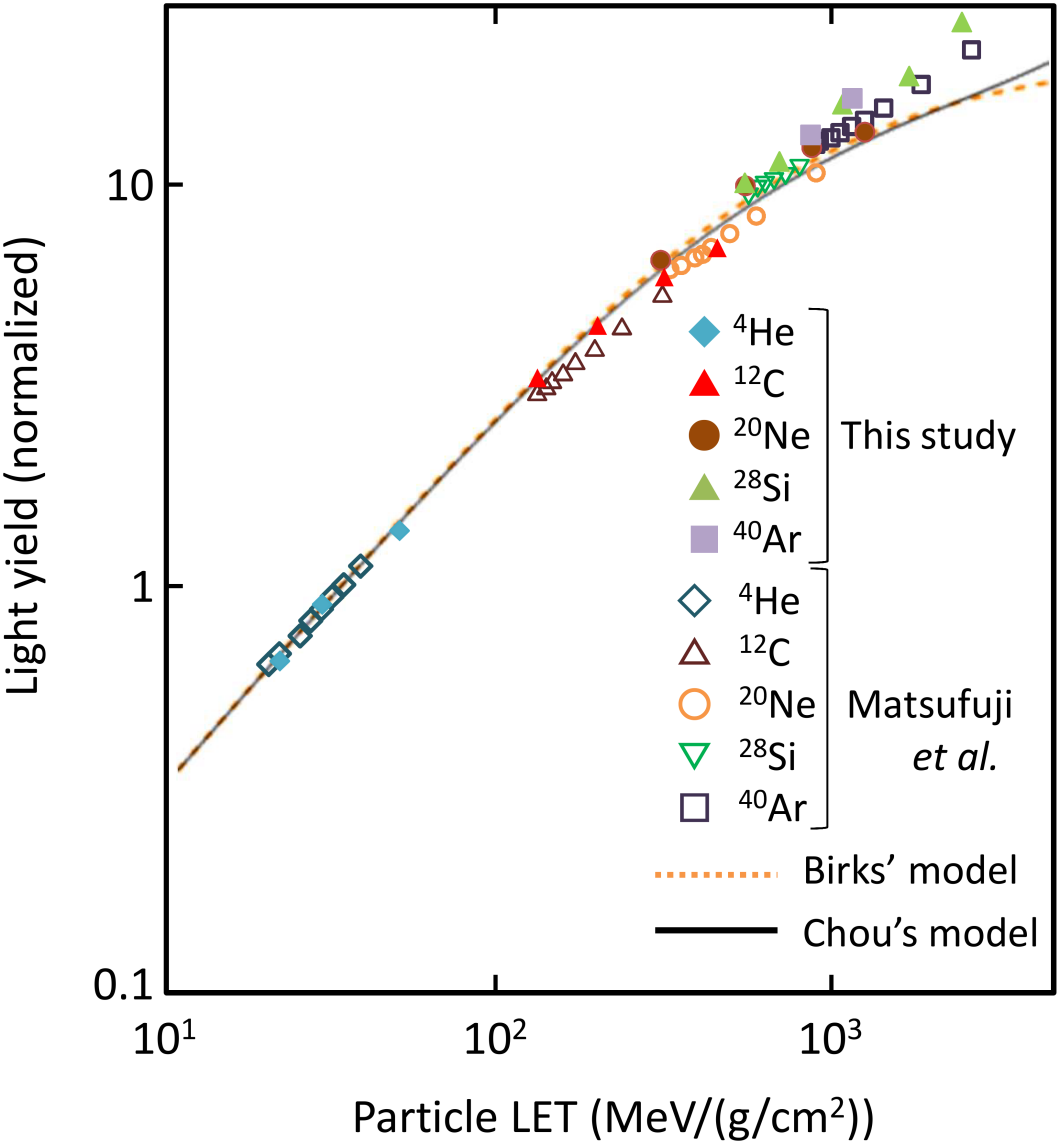
Light yield of NE-102A irradiated by energetic heavy ions (150—550 AMeV). Dashed and solid lines denote the fitting by Birks’ formula and Chou’s formula, respectively.

The light yield for high-energy ion incidence (^4^He, ^12^C, ^20^Ne, ^28^Si, and ^40^Ar from 150 AMeV to 550 AMeV) is plotted in Fig 3. Here, AMeV means MeV per projectile mass number (also written as MeV/nucleon). In this plot, the light yields were measured and calculated under the punch-through condition. All calculated data were normalized by the experimental data for 150 AMeV ^4^He at 21.7 MeV/(g/cm^2^). The light yield measured by Matsufuji [3] and that calculated by our model increase coherently with increasing LET, exhibiting a saturation trend. Except for the underestimation at 310 MeV/(g/cm^2^) (80 AMeV ^12^C), both sets of data show good agreement. The fitting by Birks’s formula and that by Chou’s formula systematically underestimate the measured light yield in high-LET region. This discrepancy suggests that these fittings have problems inherent to their formulization.

**Fig 4:**
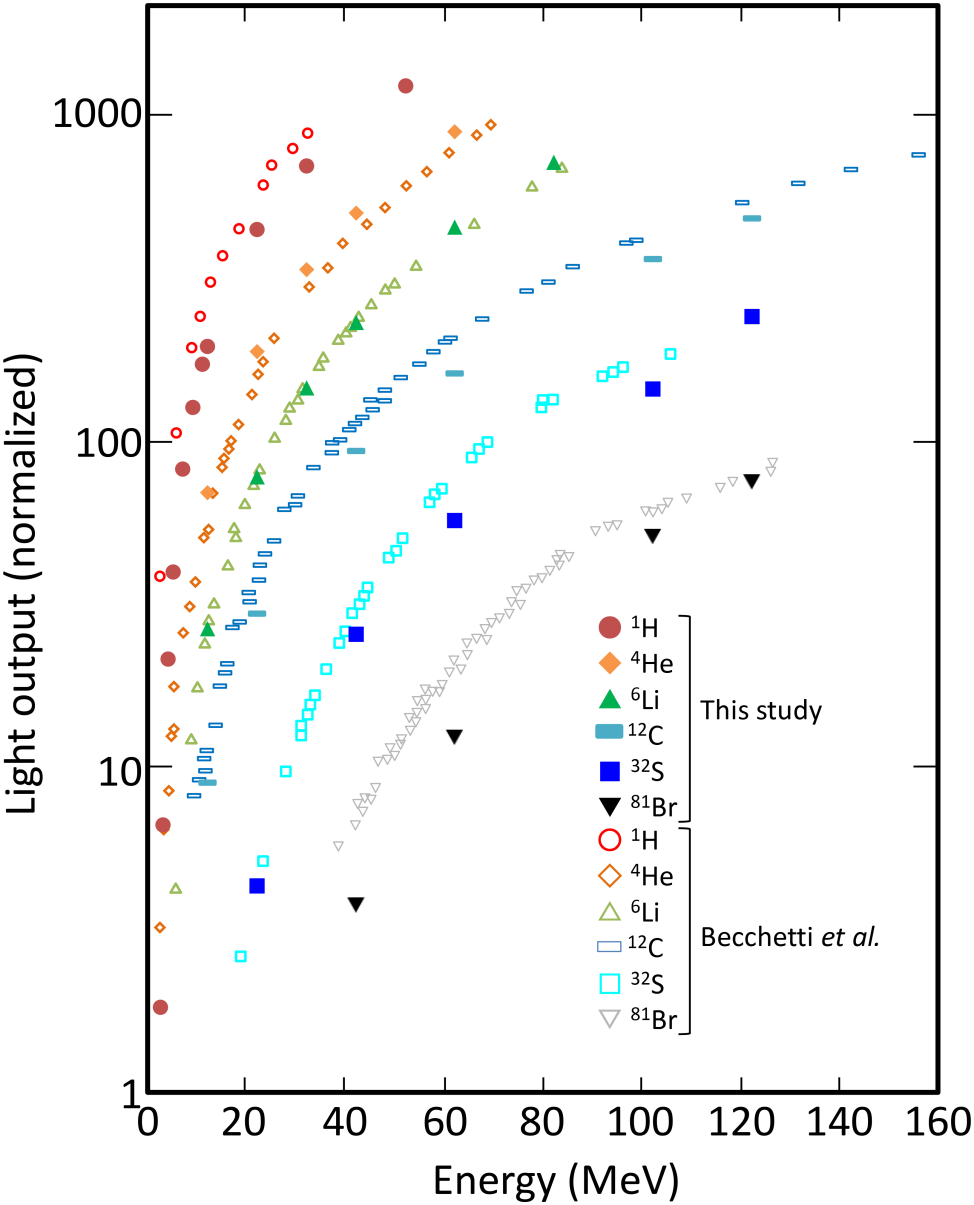
Light yield of NE-102A irradiated by low energy heavy ions (- 160 MeV).

The light yield by low-energy heavy ions obtained by Becchetti and the corresponding calculated data are shown in Fig 4. The experimental and calculated data were obtained in full-stop condition and the calculated light yields were normalized against 120 MeV ^81^Br. In this energy range, the light yield increases rapidly with incident energy. This trend is attributed to reduction of energy deposition density with increasing the incident energy, which results in weaker quenching. For the same level of energy deposition, the light yield varies by a factor of more than 10 depending on the particle species. This comparison shows that our model can reproduce such strong quenching in this energy range. The largest discrepancy found for 40 MeV ^81^Br may be partially (13%) explained by the energy deposition threshold discussed in the “Track-structure method” subsection. In fact, the number of DEA is no more than 10% of all the events but about 50% of the light yield for 40 MeV ^81^Br is attributed to DEA (not shown) because the other ionization events are concentrated around the primary ion beam whereas DEAs are caused by electrons traveling for a long distance, where interaction with other excited molecules is less likely.

**Fig 5:**
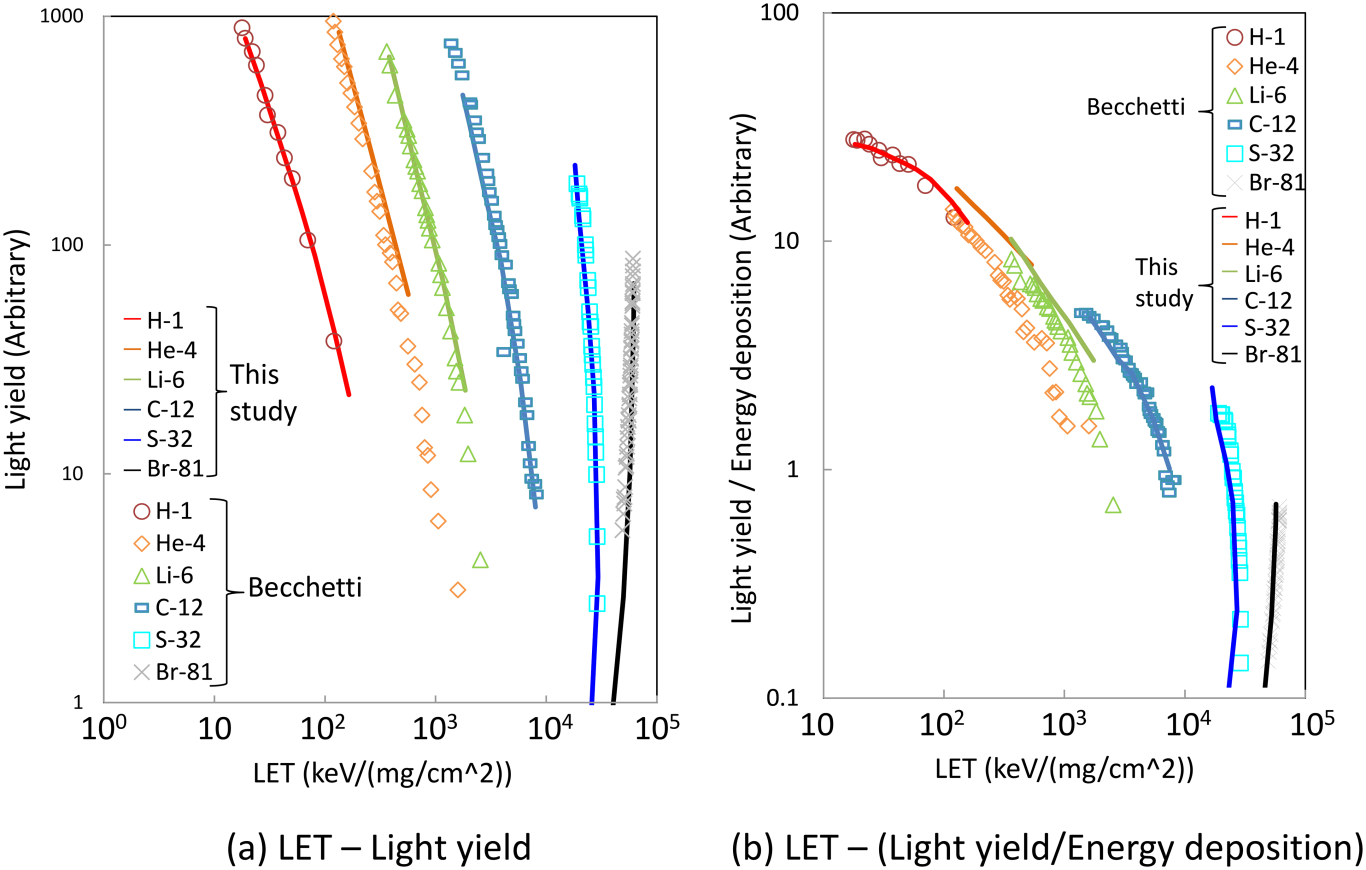
Same as Fig 4 but horizontal axis is LET. Vertical axis is (a) light yield or (b) light yield per deposited energy.

Fig 5 shows LET dependence of the light yields. By simply plotting the light yield by LET, the light yield drastically depends on the particle species in contrast to Fig 3 because the vertical axis is not the light yield per unit length as written in Eq (1). In the punch-through condition as shown in Fig 3, the thickness of light-emitting volume is always the same as the scintillator thickness whereas the length of scintillating volume is uncertain in the full-stop condition therefore the Birks’ law was modified as,

$$\begin{array}{l} \frac{dL}{dx}=S\frac{\frac{dE}{dx}}{1+kB\frac{dE}{dx}},\\ \to \frac{dL}{dE}=\frac{S}{1+kB{\left(\frac{dE}{dx}\right)}_{\text{eff}}}, \end{array}$$ (6)

to show that the Birks’ law does not hold true in this energy range. In Eq 6, ${\left(\frac{dE}{dx}\right)}_{\text{eff}}$ is effective LET. Because the higher is the LET, the lower is the contribution to light yield, LET at the upstream surface of scintillator was taken as LET in Fig 5. Fig 5(b) shows the light yield per deposited energy plotted as a function of LET. Because *k* and *B* in the Birks’ law are the parameters independent from the particle species, Eq (6) indicates that the light yield normalized by the deposited energy is a single-valued function of LET. However, the light yield owing to ^6^Li and that to heavier ions do not follow a universal trend.

**Fig 6:**
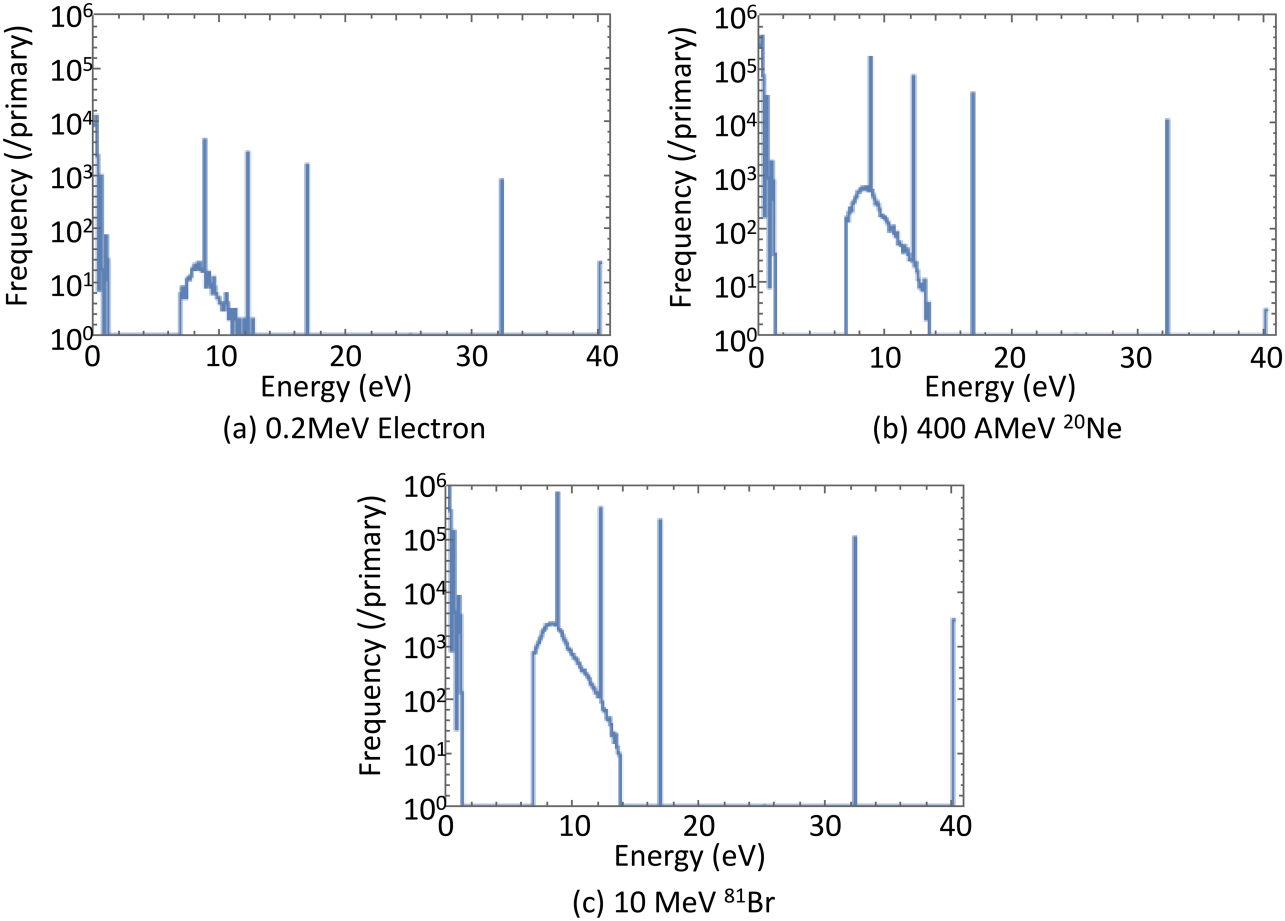
Deposition energy spectra integrated over entire volume calculated by RITRACKS.

The results discussed so far showed that the light yield owing to various particles was well reproduced by the track-structure method. Fig 6 shows that the spectra of energy deposition calculated by RITRACKS under three different irradiation conditions.

Strength of quenching was substantially different in the above-discussed conditions, however, the shapes of energy deposition spectra are independent of the incident particle species because energy deposition is attributed mainly to secondary electrons. This fact shows that quenching cannot be explained merely by calculating the energy deposition spectra but the spatial arrangements of energy deposition is indispensable to estimate the strength of quenching.

**Fig 7:**
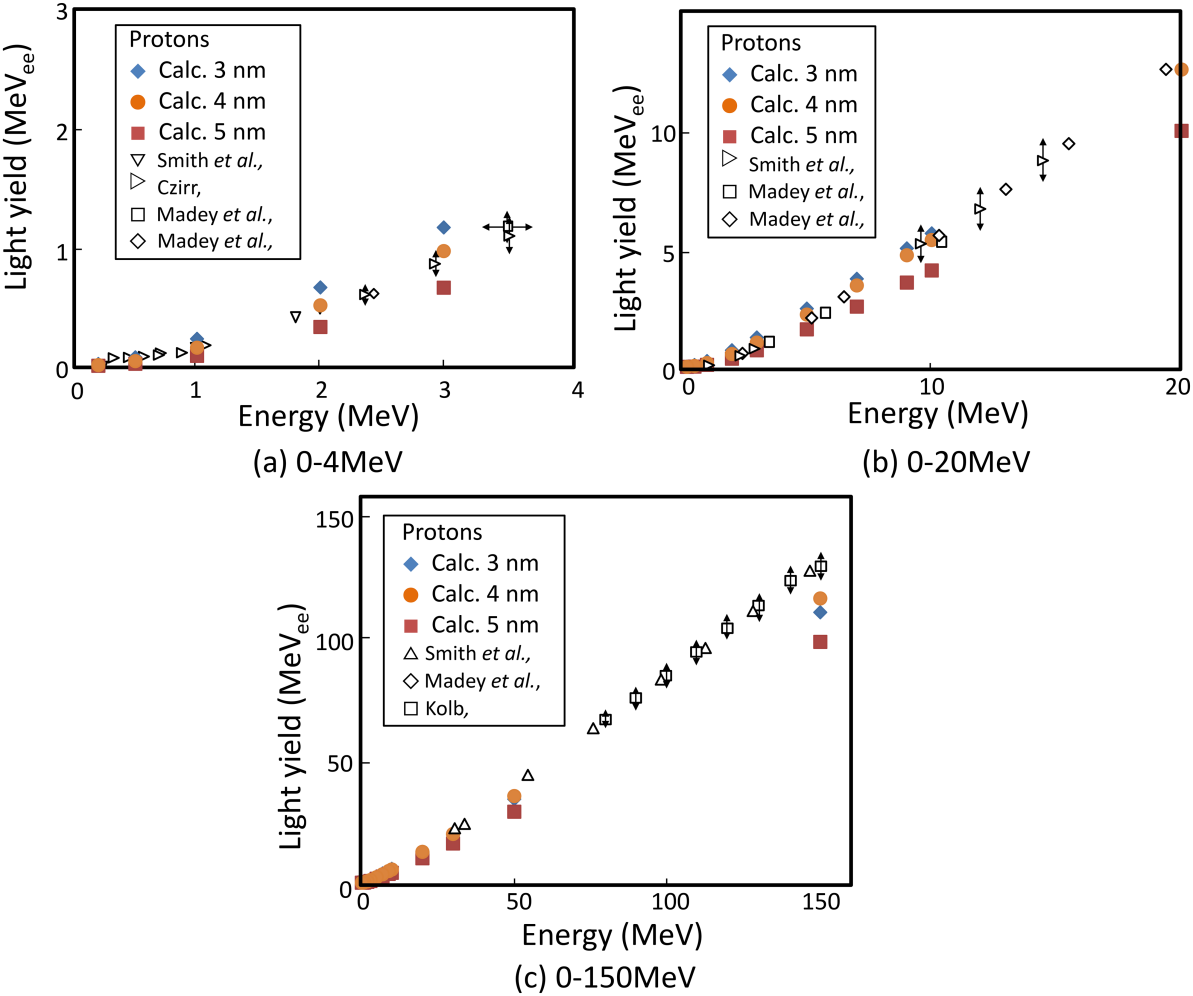
Light yields of NE-102A irradiated by protons. Calc. 3, 4, and 5 are the data calculated by track-structure method for 3, 4 and 5 nm of Förster radius, respectively. The other values are experimental data from the literature.

To examine the appropriateness of the assumed Förster radius, calculation of Fig 2 was performed with changing the Förster radius. Fig 7 shows the light yield calculated by the track-structure method for *R*_f_ = 3, 4 and 5 nm. The light yield is decreased with increase in the Förster radius owing to strong quenching. This trend is particularly pronounced for low-energy protons. At 0.5 MeV, for example, the calculated light yields for *R*_f_ = 3 nm is 3.9 times as large as that for *R*_f_ = 5 nm. This analysis indicates that FRET in NE-102A detectors depends on the Förster radius and 4 nm is a reasonable estimate to explain the experimental data.

**Fig 8:**
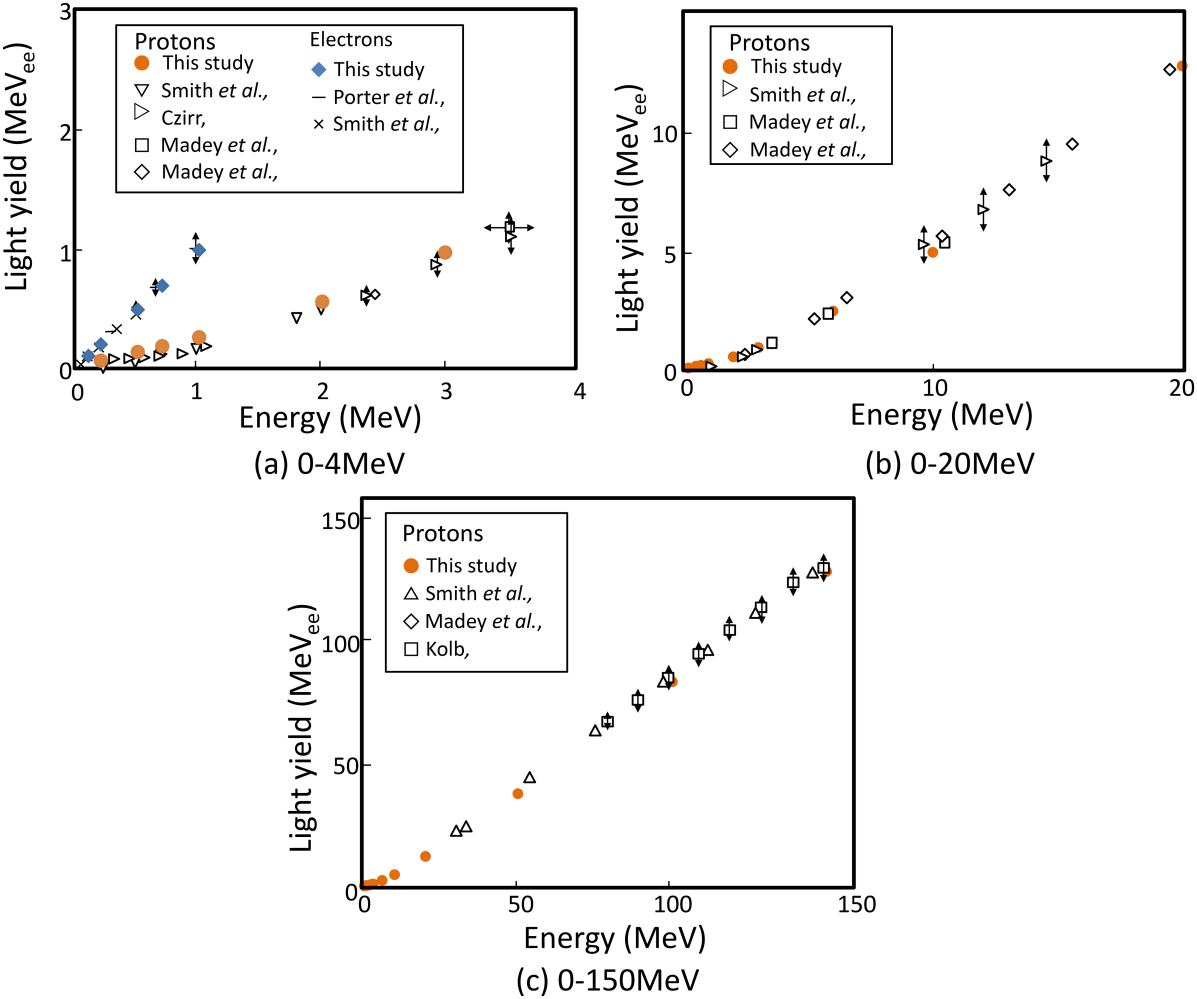
Comparison of measured and simulated light yields of NE-102A irradiated by protons and electrons at various energies. Simulation was based on the SE method.

**Fig 9:**
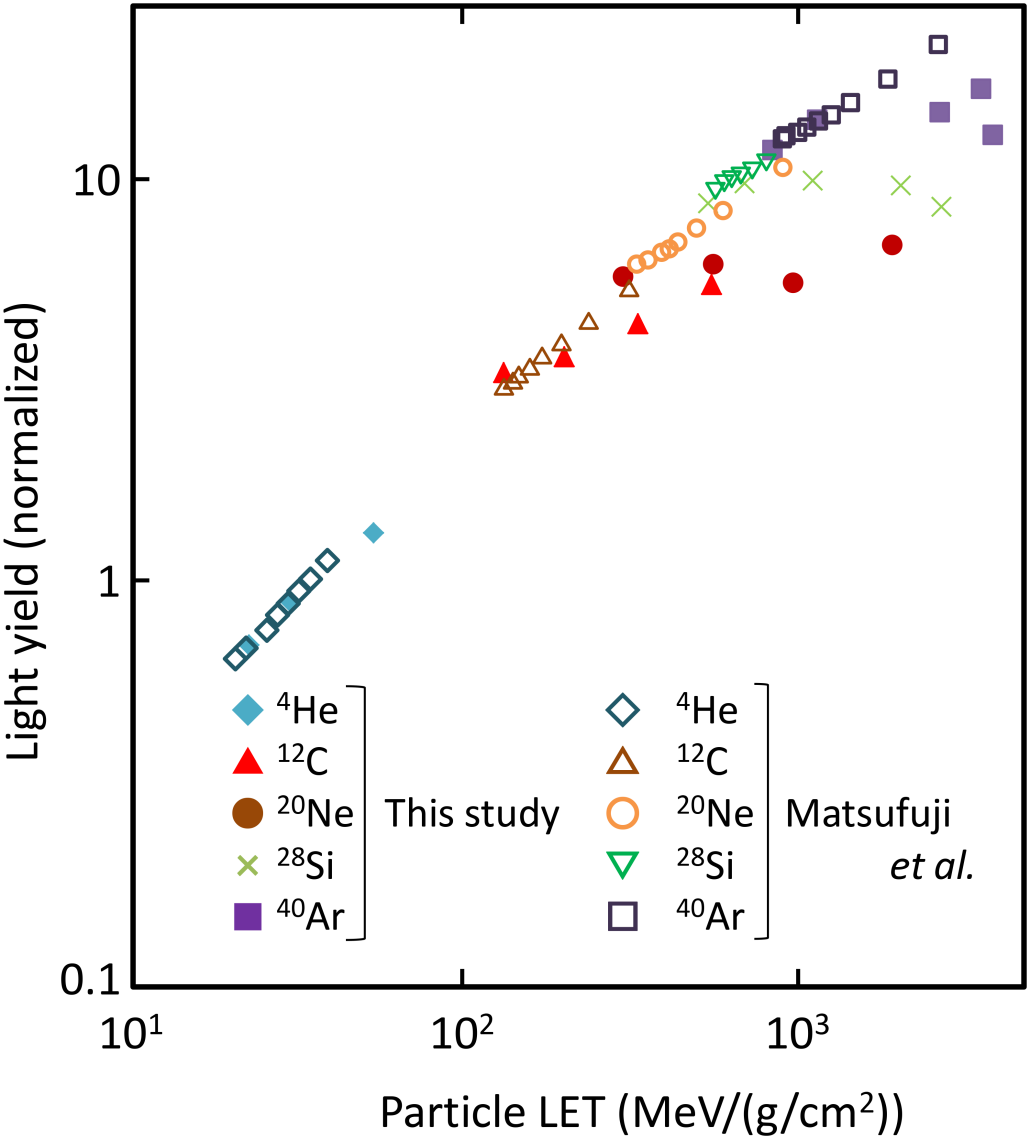
Comparison of measured and simulated light yields of NE-102A irradiated by energetic heavy ions (100—1000 AMeV). Simulation was based on the SE method.

The light yield calculated using the SE method is compared with the data from literature. Fig 8 shows a comparison of the calculated and the measured [13–18] light yields due to electrons and protons. The energy dependences of the light yield due to both electrons and protons are also reproduced by the SE method. The light yield is suppressed strongly in the case of low-energy protons owing to quenching but light yield is almost linear to deposition energy above 10 MeV. This trend agrees with that of the track structure method as seen in Fig 2. Light yield due to energetic heavy-ion incidence predicted using the SE method is plotted as Fig 9 [3, 20]. All simulated data were normalized against 150 AMeV ^4^He at 21.7 MeV/(g/cm^2^). Increase and saturation of light yield with increasing incident energy were accurately reproduced by the SE method.

**Fig 10:**
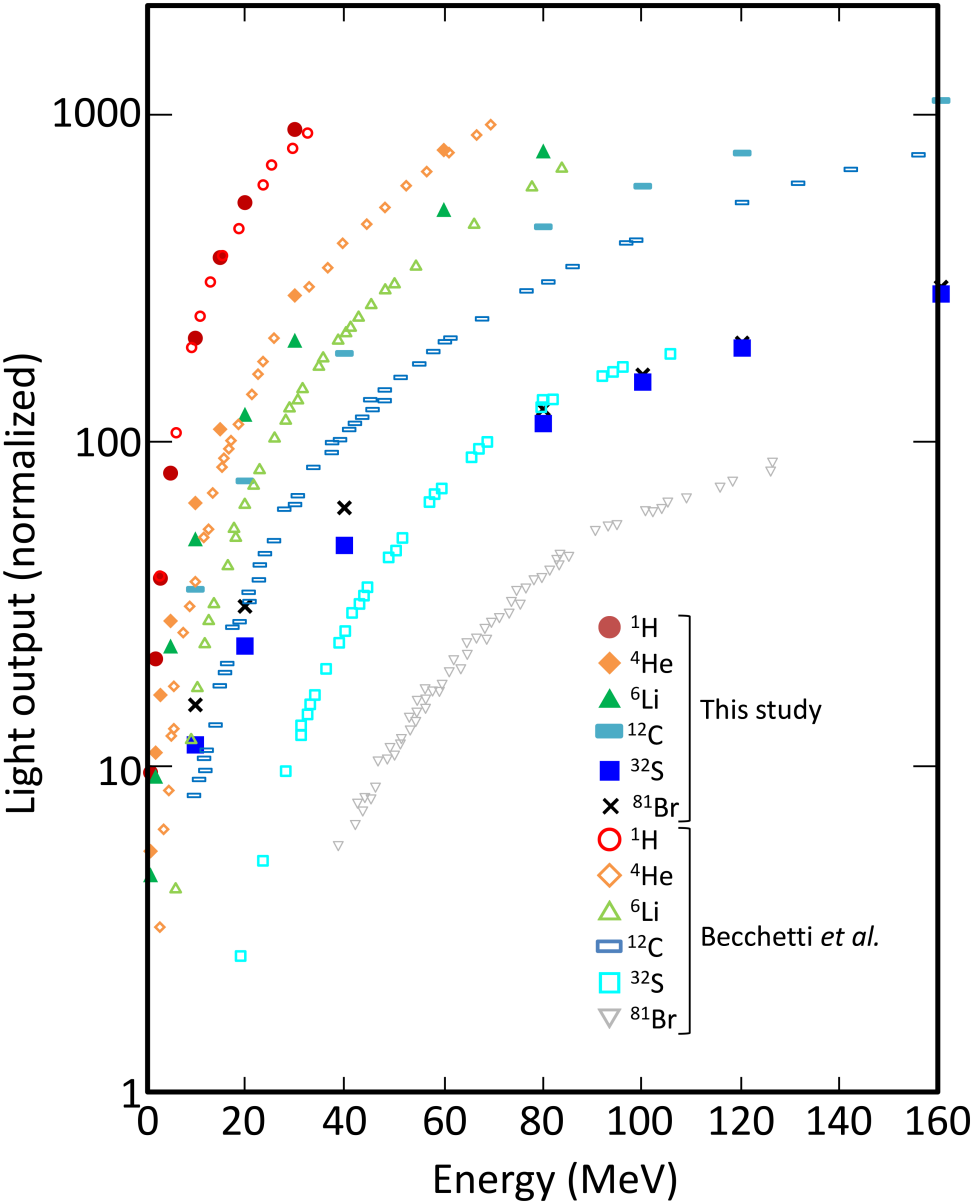
Comparison of measured and simulated light yields of NE-102A irradiated by low-energy heavy ions (- 160 MeV). Simulation was based on the SE method.

**Fig 11:**
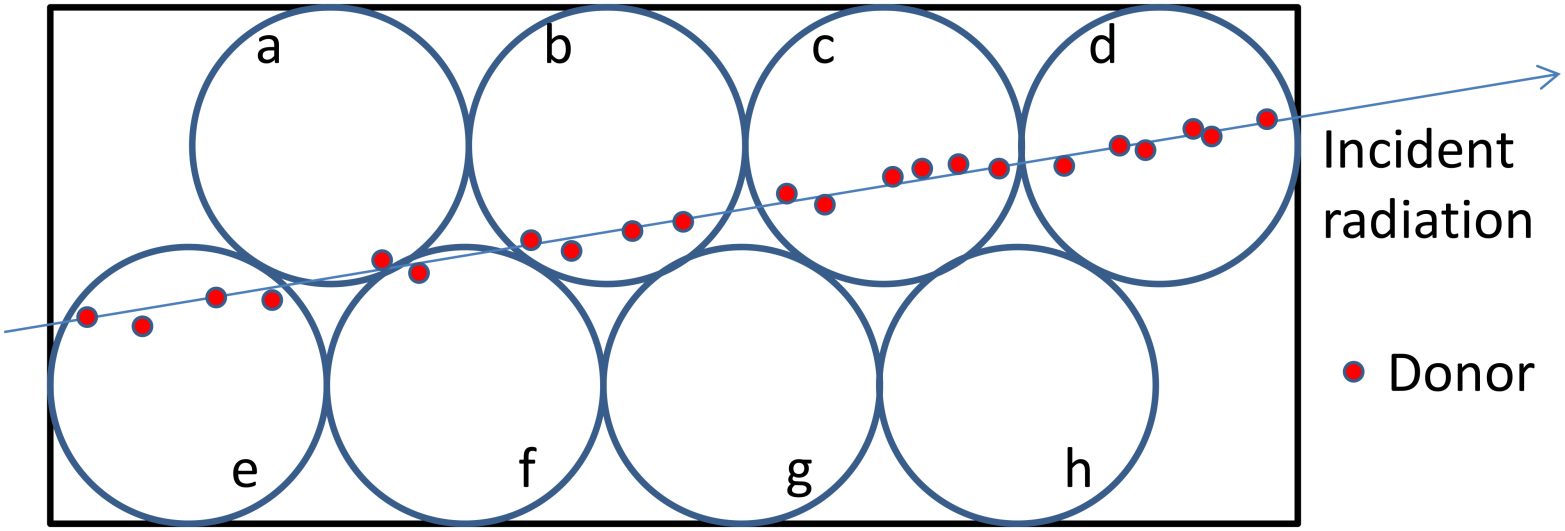
Schematic diagram of the mechanism to overestimate light yield by SE method.

In contrast, Fig 10 shows that the analysis based on the SE method does not agree with the experimental data for low-energy heavy ions. As in Fig 4, the calculated data were normalized against 2.5 MeV ^3^H. The heavier the incident ions, the greater was the deviation between the calculated data and the literature data. This deviation was attributed to the assumption adopted in the SE method which is unreasonable for high-LET particles as illustrated in Fig 11. In the SE method, the material was split into cells, and FRET between donors in neighboring cells was not considered. When the energy deposition density is large, donors are produced densely along the trajectories. However, the randomness of energy deposition and cell splitting results in cells that have a single donor near their boundaries. In reality, these donors interact with the donors in the neighboring cells but the calculation indicates that these cells contribute significantly to the light yield because quenching beyond cell boundaries was not considered. In Fig 11, cell a and f have only one donor therefore their excitation energies are not lost in the SE method, however, energy can be transferred between the donor in cell a and that in cell f and eventually lost in reality. In addition, as explained in Section “Specific energy method”, the donor distributed in each cell was approximated to be random though the spatial coordinates of donors in each cell are likely correlated particularly in high-LET particle irradiation. The overestimation of light yield may be also attributed to this approximation. Thus the SE method is inadequate for high-LET heavy ions owing to its inaccurate estimation of quenching.

Overall, the track-structure method is valid in all the conditions examined in this study. On the other hand, the SE method is valid for electrons, protons, He ions of energy greater than 20 MeV, and the other ions of energies greater than 150 AMeV. Energy deposition by heavy ions below 150 MeV/u is too dense for the SE method.

## Conclusion

We showed that the light output from NE-102A plastic scintillators exposed to electrons, protons and heavy ions can be predicted by a universal approach based on the microscopic transport of electrons considering quenching. After simulating the excitation of molecules by electrons, energy transfer from donors to other donors owing to FRET was simulated to estimate observable scintillation light yields. The simulated light yields agree with the experimental data for various particles ranging in energy from a few 100 keV to a few GeV.

Because the scintillation light yield was parameterized as a function of LET in the conventional approaches, the light yield due to low-energy light ions, the LET of which is the same as that of heavy ions at certain energies, could not be estimated. The method developed in this study, which is not based on LET, can estimate the light yield due to low-energy ions reasonably.

The production of donors was estimated either by detailed track structure simulation or by using a specific-energy calculation model. The specific-energy calculation model scheme does not consider the detailed spatial arrangement of donors, therefore track structure simulation is necessary for low-energy heavy ions. However, the specific-energy calculation model is substantially advantageous in computational time therefore they can be easily incorporated to macroscopic radiation transport simulation codes such as PHITS [11, 12] to simulate the response of detectors with considering the detector geometry, and radiation field.

This result indicates that the approach proposed in this study can be applied to other scintillators if Förster radius is known and the transport of low-energy electrons can be simulated reasonably. The prediction of light yield from scintillators will be useful for detector design, experiment planning and scintillation material development.

## Supporting information

S1 Table The numerical data of the Figs 2, 3, 4, 5 and 7. (XLSX)

S2 Table The numerical data of the Figs 8, 9 and 10. (XLSX)
